# *In vitro *analysis of the invasive phenotype of SUM 149, an inflammatory breast cancer cell line

**DOI:** 10.1186/1475-2867-5-11

**Published:** 2005-04-27

**Authors:** Michaela R Hoffmeyer, Kristin M Wall, Suranganie F Dharmawardhane

**Affiliations:** 1 Department of Molecular Cell and Developmental Biology, University of Texas Austin, 205 West 24^th ^Street, Austin, Texas, 78712, USA; 2 Department of Biomedical Engineering, University of Texas at Austin, 1 University Station Stop C0800, Austin, TX 78712, USA

## Abstract

**Background:**

Inflammatory breast cancer (IBC) is the most lethal form of locally invasive breast cancer known. However, very little information is available on the cellular mechanisms responsible for manifestation of the IBC phenotype. To understand the unique phenotype of IBC, we compared the motile and adhesive interactions of an IBC cell line, SUM 149, to the non-IBC cell line SUM 102.

**Results:**

Our results demonstrate that both IBC and non-IBC cell lines exhibit similar adhesive properties to basal lamina, but SUM 149 showed a marked increase in adhesion to collagen I. *In vitro *haptotaxis assays demonstrate that SUM 149 was less invasive, while wound healing assays show a less *in vitro *migratory phenotype for SUM 149 cells relative to SUM 102 cells. We also demonstrate a role for Rho and E-cadherin in the unique invasive phenotype of IBC. Immunoblotting reveals higher E-cadherin and RhoA expression in the IBC cell line but similar RhoC expression. Rhodamine phalloidin staining demonstrates increased formation of actin stress fibers and larger focal adhesions in SUM 149 relative to the SUM 102 cell line.

**Conclusion:**

The observed unique actin and cellular architecture as well as the invasive and adhesive responses to the extracellular matrix of SUM 149 IBC cells suggest that the preference of IBC cells for connective tissue, possibly a mediator important for the vasculogenic mimicry via tubulogenesis seen in IBC pathological specimens. Overexpression of E-cadherin and RhoA may contribute to passive dissemination of IBC by promoting cell-cell adhesion and actin cytoskeletal structures that maintain tissue integrity. Therefore, we believe that these findings indicate a passive metastatic mechanism by which IBC cells invade the circulatory system as tumor emboli rather than by active migratory mechanisms.

## Background

With an average five-year post-recovery survival rate of 45%, inflammatory breast cancer (IBC) is the most lethal and aggressive form of locally advanced breast cancer [1]. The lethality of IBC stems from its highly invasive nature. Diagnosis of IBC is often complicated by lack of a palpable precursor lesion commonly associated with breast cancer. Moreover, the correct diagnosis is hindered by inflammatory-like symptoms such as redness, warmth, and edema. Characteristic of IBC is a change in breast skin texture, similar to that of an orange, due to extensive invasion of the dermal lymphatics by IBC tumor cell emboli. These complications contribute to IBC lethality in that by the time a proper diagnosis is made, the cancer has aggressively infiltrated the surrounding tissue and lymphatics system, leading to a lowered patient prognosis [2]. Complicating treatment of this deadly form of breast cancer is that very little information about the cellular mechanisms responsible for the unique IBC phenotype is known.

Cancer cell invasion through the basal lamina and subsequent metastasis involves multiple steps including intravasation through the surrounding tissue into the lymphatic or vascular systems. Transient adhesion to extracellular matrix (ECM) components as well as modification of cell shape by reorganization of the actin cytoskeleton is required for cancer cell infiltration into the adjacent tissue. The Rho GTPases regulate actin cytoskeletal rearrangements, and are thus likely candidates for involvement in cancer cell invasion and metastasis [3,4]. Further evidence for a relationship between cancer cell mobilization and dysregulation of Rho GTPases is seen in the overexpression of Rho proteins in numerous invasive human cancers. The recent discovery of the overexpression of the Rho isoform RhoC by IBC tumors has been implicated in the physiological mechanisms of this poorly characterized form of breast cancer [5]. RhoC was demonstrated to be overexpressed in metastatic tumors of pancreatic adenocarcinoma patients [6], murine melanomas [7], and in the patient-derived IBC cell line SUM 149 [5]. Transient inhibition of RhoC in IBC cells by treatment with farnesyl transferase inhibitors reduced invasion and motility *in vitro *[8]. Recently it was reported that RhoC overexpression in mammary epithelial cells resulted in a significant increase in cell migration [9], mediated by the MAPK pathway [10]. These findings led us to hypothesize that RhoC overexpression may promote the highly invasive phenotype of IBC and contribute to the uniquely aggressive phenotype exhibited by IBC.

Another unique feature of IBC is the overexpression of E-cadherin, a transmembrane protein involved in cell-cell adhesion, which is generally lost in highly invasive cancers. It seems somewhat paradoxical that such an aggressive cancer that overexpresses proteins involved in actin cytoskeleton rearrangement and promotion of migration (i.e., RhoC) also overexpresses cell-cell junction proteins such as E-cadherin [11-15]. The literature thus far seems to hold to two schools of thought about the contradictory protein expression seen in IBC. One tends to support the idea that E-cadherin expression fluctuates with disease progression and decreases as IBC cells become invasive [15]. The second school supports the theory of passive metastasis by IBC [11,12]. In passive metastasis, strong tumor cell-cell adhesions are maintained during dissemination that proceeds via vasculogenesis through secretion of differentiation factors by the tumor cells causing *de novo *vessel formation [16]. This results in a cancer cell cluster within the vessel, reminiscent of the IBC tumor emboli seen in IBC histology. Furthermore, RhoC overexpression in human mammary epithelial cells has been shown to increase production of angiogenic factors, some of which might mediate passive or active metastasis [17].

The IBC phenotype has mystified clinicians due to its inflammatory-like symptoms. However IBC symptomology is not considered to be a true immunoreaction, but rather a consequence of cancer cell invasion to the lymphatics system. The mechanism by which IBC invades is unclear and further experimentation with IBC models is required to clarify the exact mechanism by which this form of breast cancer is disseminated. Using the SUM 149 IBC cell line, we have examined the adhesive and migratory capacities in an effort to understand the invasive behavior of IBC for future experimentation with *in situ *imaging of IBC in animal models. SUM 149 was compared to a control cell line, SUM 102, which was selected because it shares a deletion in the LIBC (lost in inflammatory breast cancer) gene with the SUM 149 cell line but reportedly expresses RhoC mRNA at low levels [5]. We show that SUM 149 is less invasive and adhesive to basal lamina components *in vitro *than SUM 102, and that SUM 149 expresses more Rho proteins and E-cadherin. These data shows that SUM 149 is not highly motile and therefore possibly not actively invasive, suggesting passive metastasis as the mechanism of IBC dissemination.

## Results

### Endogenous Levels of Rho

**Figure 1:**
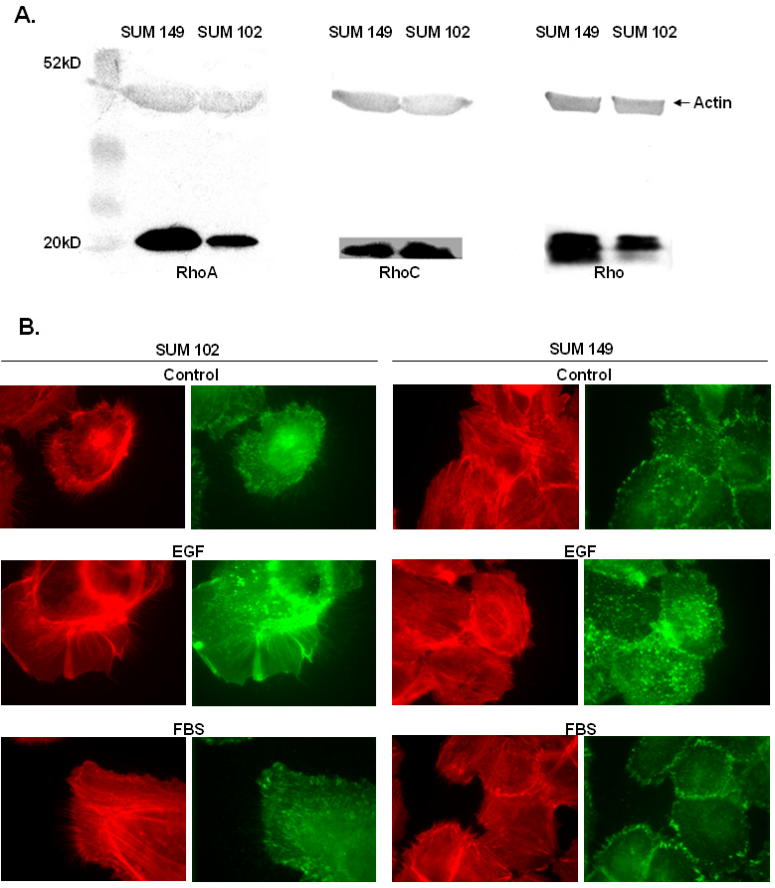
**1A. Rho GTPase protein expression levels in the IBC cell line SUM 149 versus SUM 102. 1B. F-actin and focal adhesion distribution in SUM 149 and SUM 102 human breast cancer cell lines. **1A. Equal protein amounts were separated by 10% SDS-PAGE, transferred to nitrocellulose, and probed for RhoA, RhoC, and Rho (A, B, and C). Images are representative of at least three independent experiments with actin serving to verify equal protein loading. 1B. SUM 149 and SUM 102 cells were starved in unsupplemented F-12 Hams media for 24 hours and stimulated for 10 minutes with PBS (control), EGF (50 ng/ml), or FBS (5%). Cells were stained with rhodamine phalloidin (red) to visualize F-actin and anti-phosphotyrosine (green) to visualize focal adhesion. Micrographs were taken at 1000× magnification. Images are representative for at least three independent experiments.

Figure 1A presents the relative protein levels of the various Rho isoforms in the IBC cell line SUM 149 versus the non-IBC cell line SUM 102. Previous investigators have reported overexpression of RhoC mRNA in IBC cells compared to SUM 102 [5]. To verify overexpression of RhoC at the protein level, we preformed Western blots on cell lysates using a RhoC polyclonal antibody (Santa Cruz Biotechnology, CA). We found no significant difference in RhoC protein levels between the SUM 149 and the SUM 102 cell lines. However, immunoblotting revealed a significant difference in Rho (A, B, and C), with the IBC cell line expressing much higher Rho protein levels. We then examined RhoA protein levels and found a significant overexpression of RhoA in the IBC cell line. This finding is interesting considering that RhoA has been shown to play a vital role in actomyosin-mediated contractility [21,24].

### Subcellular Distribution of Focal Adhesions and Filamentous Actin

Because RhoA is involved in actin stress fiber and focal adhesion formation, we stained the cells with rhodamine phalloidin to visualize F-actin and anti-phosphotyrosine to visualize focal adhesions. Figure 1B demonstrates F-actin and focal adhesion distribution in both cell lines. SUM 149 displayed larger focal adhesions and more actin stress fibers than the SUM 102 cell line, as might be expected from the high levels of RhoA in the SUM 149 cell line. Upon stimulation of quiescent cells with EGF or FBS, the SUM 102 cells formed large membrane ruffles (lamellipodia). However, stimulation by both EGF and FBS seemed to have little effect on the actin cytoskeleton of the SUM 149 cells. An increase in focal adhesion was seen in the SUM 149 cells after stimulation with EGF, but no clear cell polarization was observed.

### Adhesion to Extracellular Matrix Proteins

**Figure 2:**
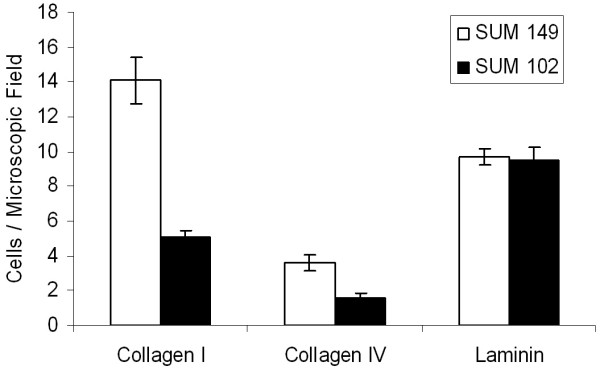
**Adhesion of human breast cancer cells to extracellular matrix proteins. **SUM 149 and SUM 102 cells (10^5^) were plated on glass coverslips coated with laminin (50 μg/ml), collagen I (10 μg/ml), or collagen IV (10 μg/ml) and allowed to adhere for 15 minutes. Micrographs were taken at 400× magnification. Adherent cells were quantified in 10 random microscopic fields. Data are expressed as mean ± SEM of at least three independent experiments.

Invasion and metastasis is a multi-step process in which cells must break local connections, move through the basal lamina, survive in circulation, and reestablish cellular attachment at distant sites. Clearly, many of these steps involve interaction with ECM components. Transient adhesion to the ECM in conjunction with cytoskeletal rearrangements are requirements for cell motility. To examine the ability of the breast cancer cell lines under study to adhere to the various ECM proteins, we performed adhesion assays (Figure 2). Here, we demonstrate that both SUM 149 and SUM 102 cells have similar adhesive properties on laminin, the major component of the basal lamina. A slight increase in adhesive properties for the IBC cells was observed compared to the SUM 102 cells on collagen IV. However, a marked increase in adhesion to collagen I, the major component of connective tissue, was seen for the SUM 149 cell line. Taken together, this data suggest that the exacerbated invasive phenotype seen in IBC is not due to differences in adhesive properties to basal lamina components, but may indicate a preference of IBC cells to the connective tissue, through which these cells must invade before entering circulation. Furthermore, attachment to connective tissue components maybe important for vasculogenic mimicry via tubulogenesis, as seen in IBC pathological specimens [25-27].

### Haptotaxis Stimulated Invasion

**Figure 3:**
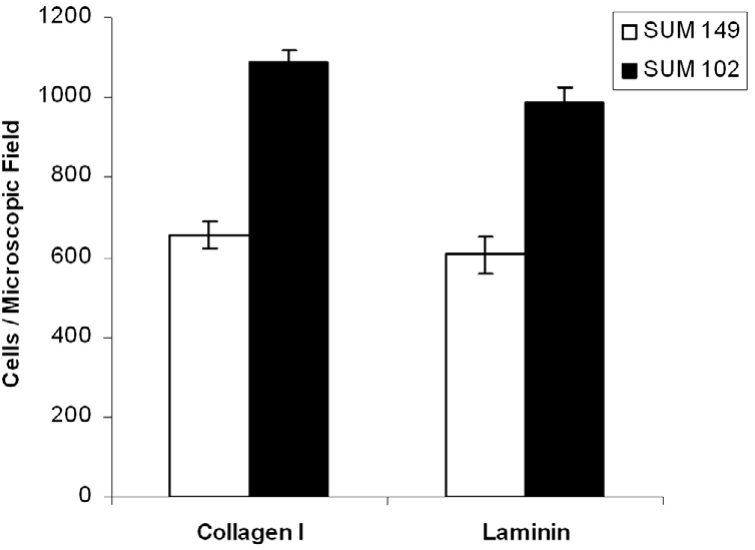
**Haptotaxis of human breast cancer cells to extracellular matrix proteins. **SUM 149 and SUM 102 cells (10^5^) were placed into the top chamber of a Costar well coated with laminin (50 μg/ml) or collagen I (10 μg/ml) and allowed to invade for 24 hours. Invasive cells were stained with propidium iodide and quantified in 10 random microscopic fields. Micrographs were taken at 200× magnification. Data are expressed as mean ± SEM of at least three independent experiments.

An aggressively infiltrative cancer must invade surrounding tissue by movement through the ECM. Haptotaxis, or cell movement toward ECM proteins, was assayed *in vitro *and is presented in Figure 3. SUM 149 cells were significantly less invasive into laminin (basal lamina component) and collagen I (connective tissue component) after 24 hours than the SUM 102 cell line. Therefore, SUM 149 was less invasive when assayed in a manner that requires individual cell movement by an active motile mechanism through a membrane with 8 μm pores.

### Subcellular Distribution of Filamentous Actin Subsequent to Cellular Polarization

**Figure 4:**
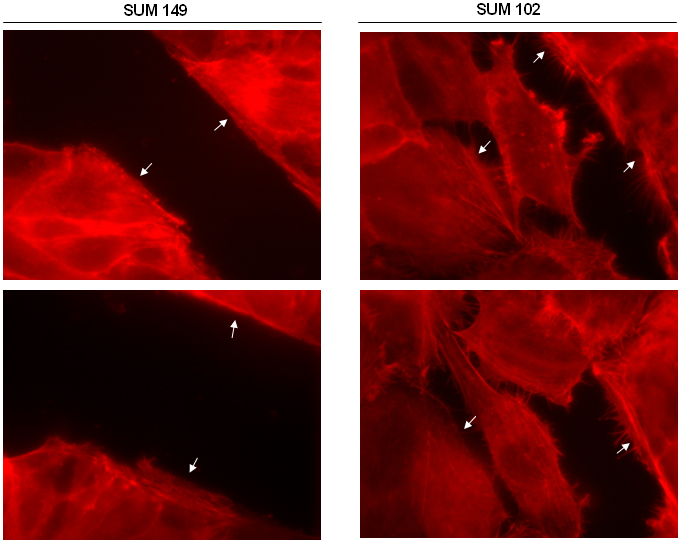
**Human breast cancer cell migration in response to wounding. **SUM 149 and SUM 102 cells were grown to confluency on glass coverslips and wounded with a sterile razor blade. Closure of the wound was monitored over 7.5 hours. Cells were stained with rhodamine phalloidin to visualize F-actin reorganization in response to cell migration. Arrows indicate the wound edge. Micrographs were taken at 1000× magnification. Images are representative of at least three independent experiments.

To induce cell polarization and migration that does not involve active migration of individual cells but rather collective cell migration over a wound edge, we performed wound healing assays as described in [28]. A confluent monolayer of cells was wounded, leading to the release of chemotractant signals by the cells at the wound edge, thus mimicking cell motility cues *in vivo *(Figure 4). Here, we present that the IBC cell line SUM 149 was less responsive to the cell-derived migration signals after 7.5 hours. By this time, the SUM 102 cell line was much more invasive into the wound space and had nearly closed the wound entirely. Thus, active migration as a sheet of cells is not likely the mechanism by which IBC is disseminated.

### Endogenous Expression of E-cadherin

**Figure 5:**
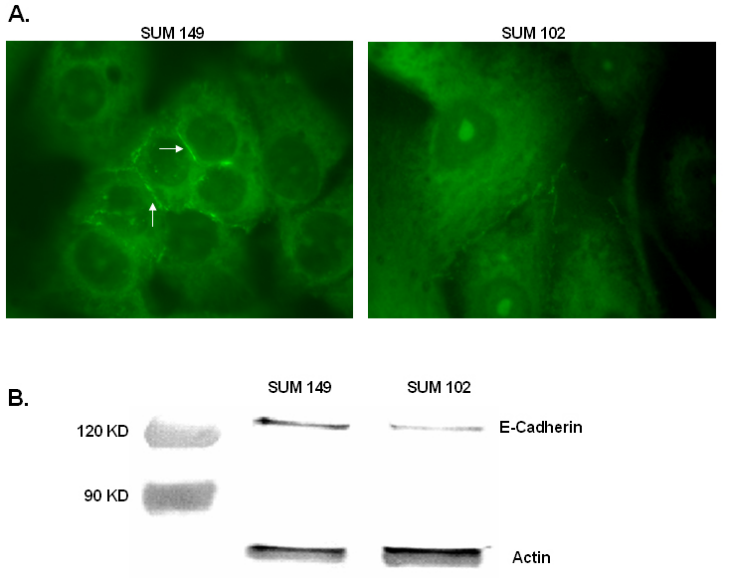
**5A. E-cadherin distribution in SUM 149 and SUM 102 human breast cancer cells. 5B. E-cadherin protein expression levels in the IBC cell lineSUM 149 versus SUM 102. **5A. Cells were grown to 60% confluency and stained with anti-E-cadherin (green). Arrows indicate cell-cell adhesions containing E-cadherin. Micrographs were taken at 1000× magnification. Images are representative for at least three independent experiments. 5B. Cell lysates were separated by 8% SDS-PAGE, transferred to nitrocellulose, and probed for E-cadherin. Images are representative of at least three independent experiments with actin serving to verify equal protein loading.

An interesting and perplexing characteristic of IBC is the expression of E-cadherin by this invasive form of breast cancer. Usually the loss of E-cadherin correlates with increased invasive and metastatic potential [29]. To verify E-cadherin expression in the IBC cell line SUM 149, we performed immunofluorescence experiments (Figure 5A). Both SUM 149 and SUM 102 show E-cadherin staining localized to the shared margins between neighboring cells, however this staining is much more intense in the SUM 149 cell line. Immunoblotting analysis likewise demonstrates E-cadherin expression by both cell lines with higher levels of E-cadherin expressed in the SUM 149 cells (Figure 5B).

## Discussion

IBC is a unique and highly aggressive form of locally advanced breast cancer with distinct clinical presentation. We hypothesized that upregulated expression of RhoC, as reported by others to be characteristic of IBC, contributes to the unusual pathological presentation of IBC. For the first time, we have compared the actin architecture, invasive, and adhesive properties of the IBC cell line SUM 149 with a cell line reported to express less RhoC mRNA compared to SUM 149 but share a deletion in LIBC [5]. Using a commercially available specific antibody to RhoC, we report that RhoC is not overexpressed at the protein level by the IBC cell line SUM 149. Interestingly, we confirmed overexpression of RhoA by utilizing an anti-RhoA specific antibody. However, post-transcriptional regulation of RhoC expression may account for the observed discrepancy. It is possible that our results do not agree with the reported mRNA expression due to specificity problems with the commercially developed antibodies. Furthermore, we demonstrate that, compared to SUM 102, SUM 149 is less invasive and migratory, and displays impaired adhesion to basal lamina components but strong adhesion to connective tissue proteins.

The role of the Rho protein in cancer cell invasion is somewhat controversial. RhoA is known to be involved in cell contractility, both in the formation of bundled actin fibers and through the activation of Rho kinase and subsequent activation of myosin light chain [30]. Such contractile cells have previously been shown to be less motile [31]. However, Rho overexpression has been documented in various human cancers such as bladder and ovarian, and correlates with lymph node invasion, metastasis, and poor patient prognosis [32,33]. Overexpression of RhoC by human mammary epithelial cells increased invasion, motility, and anchorage independent growth, similar to SUM 149 [9]. Expression of dominant negative Rho T19N has been demonstrated to block melanoma cell invasion [34]. Some investigators report that Rho overexpression has little impact on invasion and cell motility, while others demonstrate a positive correlation between Rho expression and cell migration capacity [35-37]. Rho is required for cell body contraction and tail retraction during directed cell motility, while active Rac and Cdc42 are required for lamellipodia and filopodia extension at the leading edge [30]. Thus, invasive potential is considered to be a balance between Rac, Cdc42, and Rho activities. Overexpression or activation of one of these Rho GTPases will shift this balance and result in a cellular phenotype dominated by the actin structure promoted by the activated Rho GTPase [38]. SUM 149 may display reduced invasion and migration *in vitro *compared to SUM 102 due to the overexpression of RhoA alone, thus masking the motile effects of Rac and Cdc42.

Another aspect that makes IBC so remarkable is that this form of aggressive breast cancer maintains strong E-cadherin expression [11-15]. Typically, loss of E-cadherin expression correlates with progression to metastatic disease since cancer cells must break inter-cell adhesions before attaining a motile phenotype [29]. Here, we demonstrate that the SUM 149 model of IBC maintains strong E-cadherin expression in culture, as seen in other IBC xenograft models and IBC pathological specimens. Previous reports indicate the E-cadherin axis is also complete and functional [11]. IBC histology reveals an extensive invasion of E-cadherin positive tumor cell emboli within the dermal lymphatics [11-15]. The expression of E-cadherin may be critical for invasion in that IBC is thought by some to be passively disseminated, an invasion mechanism that necessitates cell-cell attachment [12]. In this scenario, tumor cells maintain strong cell-cell connections and enter circulation via vasculogenesis around a tumor cell embolus. Others hold that E-cadherin expression varies with the malignant stage of the disease, and is lost during invasion but reestablished once tumor cells invade the vasculature [15]. The finding reported here, in which the IBC cell line SUM 149 was less invasive and adhesive *in vitro *compared to the reportedly less aggressive breast cancer cell line SUM 102, seems to support an alternative mode for IBC dissemination from classic actin cytoskeleton- mediated cell motility.

The high expression levels of both E-cadherin and RhoA by SUM 149 may contribute to the uniquely invasive phenotype of IBC. However, signaling via E-cadherin to Rho is unclear with E-cadherin-mediated Rho activation and inhibition reported in a cell line specific manner [39]. Dominant negative RhoA expression in EL and nEαCL cells has been reported to reduce E-cadherin activity [40]. During the embryonic development of stratified epithelium, it was found that α-catenin, Rho, and Rho kinase were vital for coordinated tissue movement. In this sense, cells maintain tissue architecture via cadherin binding but move as a unit through actin reorganization mediated by Rho and its downstream effector Rho kinase [41]. A parallel argument could be made for the dissemination of IBC, in which tightly bound tumor cells move as a coordinated front. This possibility was tested in a wound healing assay, in which we found that the SUM 149 cells do not polarize or move into the wound after 7.5 hours, suggesting that this form of invasion is not the mechanism by which IBC is dissemination.

## Conclusion

**Figure 6:**
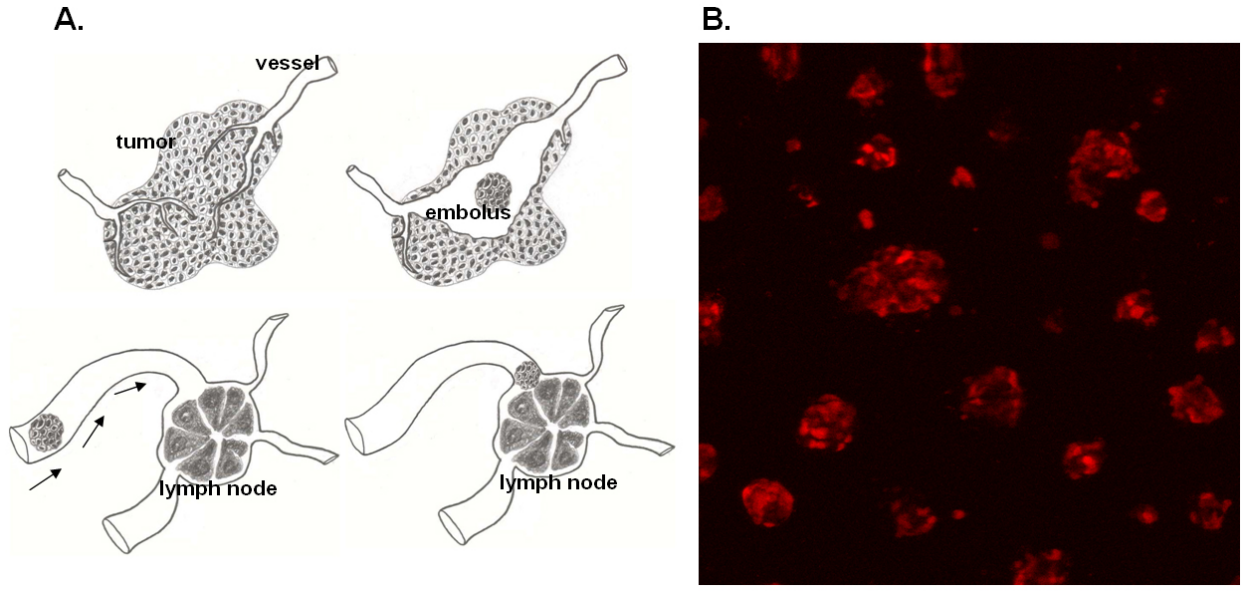
**6A. Model of passive metastasis. 6B. SUM 149 cell spheroids expressing RFP. **6A. Tumor cells secrete unknown differentiation factors, stimulating vasculogenesis and resulting in a cluster of tumor cells, termed embolus, located within the *de novo *formed vessel. The embolus maintains cell-cell attachments as it moves through the vessel and lodges within a dermal lymph node. 6B. 100× confocal projection image (10 images, Z = 20 μm) of SUM 149 cell spheroids stably expressing RFP in 3-dimensional matrigel culture after 5 days.

Thus, our results demonstrate that the IBC cell line SUM 149 is less invasive than a similar cell line, SUM 102, which expresses less Rho. This finding seems to support an alternate mode of dissemination for IBC than that of the classic invasive model, in which individual cells break local attachments and move through the ECM via actin cytoskeleton remodeling. A previously hypothesized mode of invasion for IBC, termed passive metastasis, would then seem the likely candidate (Figure 6A). In passive metastasis, vasculogenesis, stimulated by secreted differentiation factors, occurs around a tumor cell embolus that has maintained strong cell-cell attachments [16]. IBC is known to secrete angiogenic and possibly also vasculogenic growth factors, such as VEGF, bFGF, IL-6, and IL-8 [17]. Vasculogenic tubule formation by melanoma cells has been shown to be dependent on cadherin expression [42]. E-cadherin positive tumor cell emboli located within the dermal lymphatics are typically found in IBC histological specimens [15]. Three-dimensional culture of SUM 149 cells in matrigel results in IBC cell spheroids that are reminiscent of the tumor cell emboli seen in pathology (Figure 6B). The probability that IBC cells invade the circulatory system by a passive metastasis mechanism as tumor emboli rather than by active migratory mechanisms is being tested by *in vivo *image analysis of fluorescent protein tagged SUM 149 mammary tumors in SCID mice. This investigation could drastically change the course of IBC treatment and identify new therapeutic targets specific for this form of breast cancer.

## Methods

### Cell Culture

The SUM cell lines used for the study have been recently developed from pleural effusions of breast cancer patients [18,19] and are generous gifts of Dr. Stephen Ethier, The University of Michigan, MI. SUM 149 is an IBC cell line that lacks expression of the gene LIBC and overexpresses RhoC [5]. SUM 102, developed from a minimally invasive human breast carcinoma [20] will be used as a model for non-IBC human breast cancer cells. SUM 149 cells were cultured in F-12 Hams (Gibco™, CA) supplemented with 5% fetal bovine serum (Tissue Culture Biologicals, CA), insulin, and hydrocortisone. SUM 102 cells were cultured in F-12 Hams (Gibco™, CA) supplemented with 5% bovine serum albumin (BSA), epidermal growth factor, T3, ethanolamine, and sodium selenite.

### Adhesion Assays

Cell adhesion assays were performed according to [21]. Briefly, glass coverslips (Fisher Scientific, TX) were coated with 50 μg/ml laminin (Gibco BRL, MD), 10 μg/ml of collagen I (BD Biosciences, MA), 10 μg/ml of collagen IV (BD Biosciences, MA) and incubated overnight at 4°C. The coverslips were blocked for 1 hour with 1% heat-denatured BSA (Sigma Chemical Corporation, MO) in PBS. Cells (10^5^) were placed on coverslips and allowed to adhere for 15 minutes. Non-adherent cells were removed by washing. The adherent cells were fixed in 3.7% formaldehyde (Sigma Chemical Corp., MO) and stained for F-actin as described below to aid in quantification. The number of cells per coverslip was quantified with a 40× phase contrast objective.

### Haptotaxis Invasion Assay

Cell invasion assays were performed as described in [22]. Modified Boyden chambers (tissue culture treated, 6.5 mm diameter, 10 μm thickness, 8 μm pores, Transwell^®^, Costar Corp., Cambridge, MA) were coated on the upper surface (invasion), of the membrane with 50 μg/ml laminin, 10 μg/ml collagen I, or 10 μg/ml collagen IV overnight at 4°C and then placed into the lower chamber containing 500 μl culture media with 10% fetal bovine serum (FBS). Serum starved cells (10^5^) were added to the upper surface of each migration chamber and allowed to migrate to the underside of the membrane for 24 hours (invasion). The non-migratory cells on the upper membrane surface were removed with a cotton swab, and the migratory cells attached to the bottom surface of the membrane stained with propidium iodide (CalBioChem-Novabiochem Corp., CA). The number of invasive cells per membrane was counted with an Olympus upright fluorescence microscope with a 40× objective.

### Wound Healing Assay

Cells were first grown to a confluent monolayer, wounded with a sterile razor blade and allowed to migrate for 7.5 hours before fixing, permeabilizing, and blocking. Cells were then stained for F-actin as described below and visualized using an Olympus upright fluorescence microscope.

### Immunofluorescence Microscopy

For focal adhesion and F-actin staining, cells were cultured on coverslips until they reached 60% confluency and starved for 24 hours in unsupplemented F-12 Hams. Cells were then stimulated with 50 ng/ml epidermal growth factor (EGF), 5% FBS, or PBS control for 10 minutes, fixed in 3.7% formaldehyde (Sigma Chemical Corp., MO), permeabilized with 0.2% Triton X-100 (Sigma, MO), and blocked with 5% goat serum (Gibco™, CA), and 5% BSA (Sigma Chemical Corp., MO) in PBS. Cells were stained with rhodamine phalloidin (Molecular Probes Inc., OR) to visualize F-actin, and a mouse monoclonal anti-phosphotyrosine antibody, clone 4G10 (Upstate Biotechnology, NY), followed by FITC-conjugated goat anti mouse IgG (ICN Biomedicals Inc., CA) to visualize the focal adhesions. Phosphotyrosine staining to is commonly utilized to visualize focal adhesions [23]. For E-cadherin staining, cells were cultured until 60% confluency, fixed in methanol at -20°C for 15 minutes, and blocked with 5% goat serum (Gibco™, CA) and 5% BSA (Sigma Chemical Corp., MO) in PBS. Cells were stained with a mouse monoclonal anti-E-cadherin antibody, clone G-10 (Santa Cruz Biotechnology, CA) followed by FITC-conjugated goat anti-mouse IgG (ICN Biomedicals Inc., CA). Cells were imaged using an Olympus upright fluorescence microscope with Spot Advanced digital camera software, Version 2.2.1 (Diagnostic Instruments Inc., MI).

### Immunoblotting

Cells were cultured to confluency on 6 cm plates, trypsinized and the pellet washed in 1X PBS. The cell pellet was then lysed in 1% NP-40 lysis buffer. Equal amounts of protein, as determined by Bio-Rad (Hercules, CA) total protein assay, were then separated by 10% SDS-PAGE gel for Rho (A, B, and C), RhoA, and RhoC, or 8% SDS-PAGE gel for E-cadherin. Cellular proteins were then transferred to a nitrocellulose membrane. Membranes were blocked with 4% milk 0.05% Tween and probed with rabbit polyclonal anti-Rho (A, B, and C) (Upstate Biotechnology, NY), mouse monoclonal anti-RhoA (Santa Cruz Biotechnology, CA), goat polyclonal anti-RhoC (Santa Cruz Biotechnology, CA), or mouse monoclonal anti-E-cadherin (Santa Cruz Biotechnology, CA) followed by horseradish peroxidase-conjugated goat anti-mouse antibody (Pierce Endogen, IL) for Rho or alkaline phosphatase conjugated goat anti mouse antibody for E-cadherin (Pierce Endogen, IL). Rho immunoblots were detected with the Super Signal West Femto-Substrate chemiluminescence kit (Pierce Endogen, IL) and Kodak Biomax MR film (Fisher Scientific, TX). E-Cadherin immunoblots were detected with NBT/BCIP alkaline phosphatase substrate (Pierce Endogen, IL).

## Competing interests

The author(s) declare that they have no competing interests.

## Authors' contributions

MRH participated in design of the study, carried out the assays, including their quantification and interpretation, and was the primary author of the manuscript. KMW participated in the cell culture, assisted in the assays and their quantification and interpretation, and helped to draft and revise the manuscript. SFD was responsible for the conception of the project, advise and training on experimental design and procedures, aided in the analysis and interpretation of data, and helped revise the manuscript. All authors read and approved the final manuscript.
